# Tantalum implanted entangled porous titanium promotes surface osseointegration and bone ingrowth

**DOI:** 10.1038/srep26248

**Published:** 2016-05-17

**Authors:** Qi Wang, Yuqin Qiao, Mengqi Cheng, Guofeng Jiang, Guo He, Yunsu Chen, Xianlong Zhang, Xuanyong Liu

**Affiliations:** 1 Department of Orthopedics, Shanghai Sixth People’s Hospital, Shanghai Jiao Tong University, Shanghai 200233, China; 2 State Key Laboratory of High Performance Ceramics and Superfine Microstructure, Shanghai Institute of Ceramics, Chinese Academy of Sciences, Shanghai 200050, China; 3 State Key Lab of Metal Matrix Composites, School of Materials Science and Engineering, Shanghai Jiao Tong University, Shanghai 200240, China

## Abstract

Porous Ti is considered to be an ideal graft material in orthopaedic and dental surgeries due to its similar spatial structures and mechanical properties to cancellous bone. In this work, to overcome the bioinertia of Ti, Ta-implanted entangled porous titanium (EPT) was constructed by plasma immersion ion implantation & deposition (PIII&D) method. Ca-implanted and unimplanted EPTs were investigated as control groups. Although no difference was found in surface topography and mechanical performances, both Ca- and Ta-implanted groups had better effects in promoting MG-63 cell viability, proliferation, differentiation, and mineralization than those of unimplanted group. The expression of osteogenic-related markers examined by qRT-PCR and western blotting was upregulated in Ca- and Ta-implanted groups. Moreover, Ta-implanted EPT group could reach a higher level of these effects than that of Ca-implanted group. Enhanced osseointegration of both Ca- and Ta-implanted EPT implants was demonstrated through *in vivo* experiments, including micro-CT evaluation, push-out test, sequential fluorescent labeling and histological observation. However, the Ta-implanted group possessed more stable and continuous osteogenic activity. Our results suggest that Ta-implanted EPT can be developed as one of the highly efficient graft material for bone reconstruction situations.

Along with the popularization of orthopedic procedures, such as knee or hip arthroplasy, the amount of revision surgeries is booming in recent years^1^,^2^,^3^,^4^,^5^. Severe bone defects usually came as the most difficult challenge in these revision cases. To deal with it, metal augment is commonly used as graft material. Titanium (Ti) and its alloys have been used as implants for the favorable mechanical features and good biocompatibility. However, implantation of Ti prosthesis frequently causes bone atrophy and reabsorption due to the high elastic modules of Ti over 100 GPa^6^. In contrast, spatial structures and mechanical properties of the porous Ti are similar to those of cancellous bone, thus providing great potential for bone reconstruction^7^,^8^,^9^. Beside the physical advantages, to be applicable bone substitutes or bulk structural materials, porous Ti also requires sufficient abilities in inducing surface osseointegration around implant and bone growth into the inner pores of materials. However, the inherent bioinertia of Ti cannot meet these requirements^10^,^11^. Therefore, surface modifications with some bioactive ions, such as calcium (Ca)^12^, zinc (Zn)^13^,^14^, hafnium (Hf)^15^, and strontium (Sr)^16^, have been applied to improve the biological performances of porous Ti^17^,^18^,^19^.

Among the various surface modification techniques, plasma immersion ion implantation & deposition (PIII&D), which has been thought to be a non-line-of-sight method particularly suitable for biomedical products with complex spatial structures^20^,^21^,^22^, is an effective method to improve the osteogenic activity by implanting osteoinductive elements into the surface layers of base materials^23^,^24^,^25^,^26^. In our previous research, Ca modified Ti by PIII&D efficiently promoted osteoblasts adhesion, proliferation, maturation, mineralization, and new bone formation in early times^27^. Ca implanted Ti materials also has beneficial effects in promoting biocompability, oxygen affinity, and osseointegration^28^,^29^,^30^,^31^. However, the Ca cathode is sensitive to the atmosphere and suffers from poor stability. This will cause storage and usage problems in quality management for future medical manufacture. These concerns, therefore, raise the prospect that other stable but active elements seemed attractive as options.

The stable chemical element Tantalum (Ta) can stably exist in the surface layers of base materials. The stable Ta_2_O_5_ protective film can provide better corrosion resistance than that of TiO_2_ film^32^,^33^. Interestingly, Ta is one of the promising materials in promoting surface osseointegration and bone ingrowth^32^,^34^,^35^,^36^. The formation of Ta–OH groups can facilitate the adsorption of calcium and phosphate ions, thus enhancing osteoblasts adhesion, proliferation, and differentiation and osseointegration^37^,^38^,^39^. However, Ta has obvious shortage on strength bearing^40^, and the large modulus over 186GPa and density about 16.6 g/cm^3^ make it hard to use as orthopedic implant on clinic^34^.

In this study, Ta implanted entangled porous Ti (Ta-implanted EPT), that combined both the advantages of Ti and Ta, was constructed by PIII&D method. Through comparing with the unimplanted entangled porous Ti (Unimplanted EPT) and Ca implanted entangled porous Ti (Ca-implanted EPT), the osteogenic properties of Ta-implanted EPT in promoting surface osseointegration and bone ingrowth were evaluated in the study. The osteogenic activity of different EPTs was evaluated by a series of *in vitro* experiments using MG-63 cells. Through using the rabbit femur implantation models, the osteogenic activity was examined by sequential fluorescent labeling, and the new bone formation around implants and inside the pores of EPT was determined using micro-CT, optical microscopy and back scattered scanning electron microscopy (SEM). Furthermore, the fixation strength between implant and femur was evaluated by push-out tests.

## Results

### Sample surface topography and characterization of EPTs

**Figure 1:**
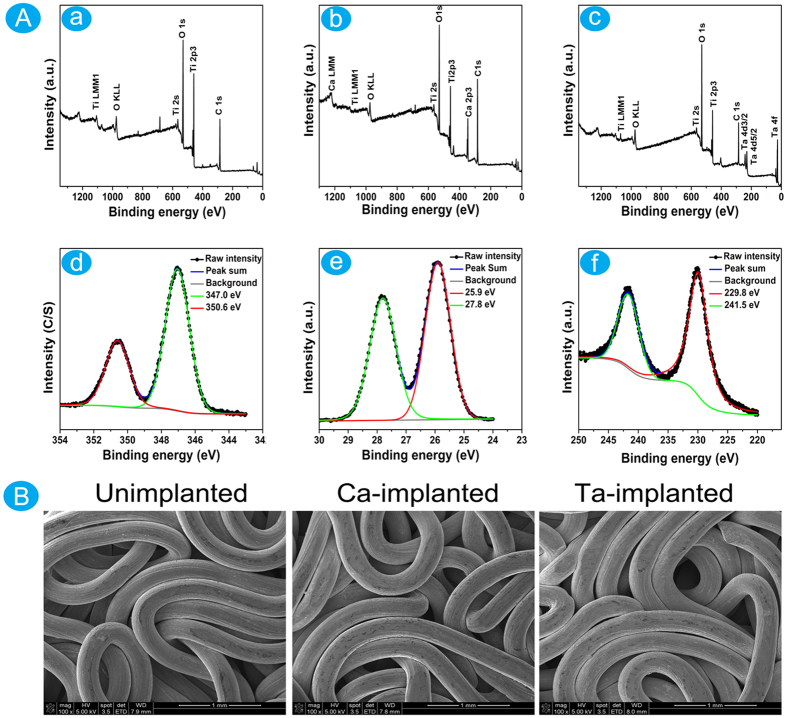
(**A**) XPS full spectra analyses of materials: (a) Unimplanted EPT; (b) Ca-implanted EPT; (c) Ta-implanted EPT. XPS high resolution spectra of surface layers: (d) Ca-implanted EPT; (e,f) Ta-implanted EPT. (**B**) SEM images of surface morphology of the Unimplanted, Ca- and Ta-implanted EPTs.

Three kinds of EPT samples were successfully prepared for this study. Just as the XPS survey spectrum shown, Ca or Ta was not discovered on the surface of unimplanted EPT (Fig. 1Aa). Except for the characteristic peaks of Ti, O and C, the feature peaks of Ca were found on the surface of Ca-implanted EPT (Fig. 1Ab), and the content of Ca reached 17.95% (Table 1). The feature peaks of Ta suggested the successful construction of Ta-implanted EPT (Fig. 1Ac), and the content of Ta was 10.96% (Table 1).

In the high-solution spectrum (Fig. 1Ad), the typical double peaks Ca 2p3/2 and Ca 2p1/2 at 347.0 eV and 350.6 eV suggested that the calcium oxide on the titanium surface reacted with ambient water to form calcium hydroxide (H cannot be detected by XPS), and then calcium hydroxide further reacted with carbon dioxide in air to form CaCO_3_^41^,^42^. The characteristic peaks of Ta 4f5/2 (25.9 eV), Ta 4f7/2 (27.8 eV), Ta 4d5/2 (229.8 eV), and Ta 4d3/2 (241.5 eV) were detected from the surface layer of Ta-implanted EPT (Fig. 1Ae,f). The peaks of Ta 4f5/2 and Ta 4f7/2 were consistent with the typical binding energy of Ta_2_O_5_^43^, and this suggested that the Ta ion on the surface of Ta-implanted EPT was oxidized after exposure to air^23^. What’s more, the SEM images suggested that no difference existed in surface topography among three kinds of materials (Fig. 1B). The PIII&D treatment did not affect the porosity of EPT materials.

In addition, no difference was discovered in compressive yielding stress and Young’s modules (Table 2). And these suggested the high similarity of unimplanted, Ca- and Ta-implanted EPTs in mechanical performances.

### Influences of different EPT materials on MG-63 cell proliferation and differentiation

**Figure 2:**
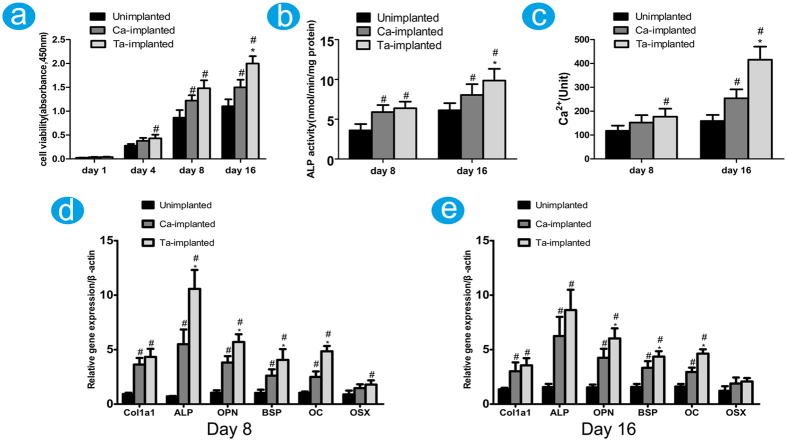
Histograms showing the cell viability (**a**), ALP activity (**b**), and the intracellular Ca ions level in MG-63 cells (**c**) after incubation with Unimplanted, Ca- or Ta-implanted EPTs at designed times, respectively. (**d,e**) The expression level of osteogenic-related markers at day 8 and 16 was measured by qRT-PCR, respectively. ^#^(p < 0.05) when compared with Unimplanted group; *(p < 0.05) when compared with Ca-implanted group.

MG-63 cells were used to evaluate the influences of different EPTs on cell viability, proliferation, and differentiation. Results of CCK-8 assay showed that both Ca- and Ta-implanted EPTs had positive effect in promoting osteoblast viability compared with unimplanted EPT after incubation for 8 and 16 days. Notably, Ta-implanted EPTs exhibited the highest effect on day 16 (Fig. 2a). The ALP activity of both Ca- and Ta-implanted groups was significantly higher than that of unimplanted group after incubation for 8 and 16 days (Fig. 2b). More importantly, Ta-implanted group had the highest ALP activity after incubation for 16 days. Similarly, the intracellular Ca density of Ca- and Ta-implanted groups was significantly higher than that of unimplanted group on day 8 and 16, but Ta-implanted group had the highest concentration of Ca ions on day 16 (Fig. 2c). These results suggested that both Ca- and Ta-implanted EPTs have favorable effects in promoting osteoblast viability, proliferation, differentiation, and mineralization of extracellular matrix (ECM) compared with unimplanted EPT, but Ta-implanted EPT has more stable and continuous effects than Ca-implanted EPT.

**Figure 3:**
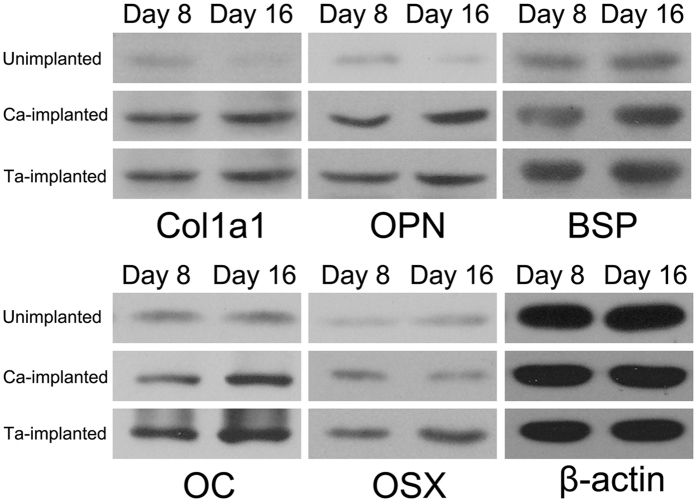
The expression of osteogenic-related markers in MG-63 cells was examined by western blotting after incubation with different EPTs for 8 and 16 days.

The expression of osteogenic-related markers, Col1a1, ALP, OPN, BSP, OC, and OSX was examined by qRT-PCR at day 8 and 16, respectively (Fig. 2d,e). Both Ca- and Ta-implanted groups had significantly higher expression level of most genes than the unimplanted group. And Ta-implanted group exhibited the highest expression level, especially for gene ALP, OPN, BSP, and OC. In addition, the results of western blotting were almost consistent with those of qRT-PCR (Fig. 3). The expression of protein Col1a1, OPN, BSP, OC, and OSX of both Ca- and Ta-implanted groups was significantly higher than that of unimplanted group. Notably, Ta-implanted EPT had the best effect in promoting the expression of protein BSP, OC, and OSX. These genes and proteins, which are regarded as the early or later markers of osteogenic differentiation, play important roles in promoting bone formation *in vivo*. Therefore, both Ca- and Ta-implanted EPTs can efficiently promote the differentiation of osteoblasts and mineralization of ECM. More importantly, Ta-implanted EPT has more stable and continuous effects than Ca-implanted EPT in the long term.

### *In vivo* osseointegration and bone ingrowth evaluation

#### Micro-CT assessment about surface osseointegration

**Figure 4:**
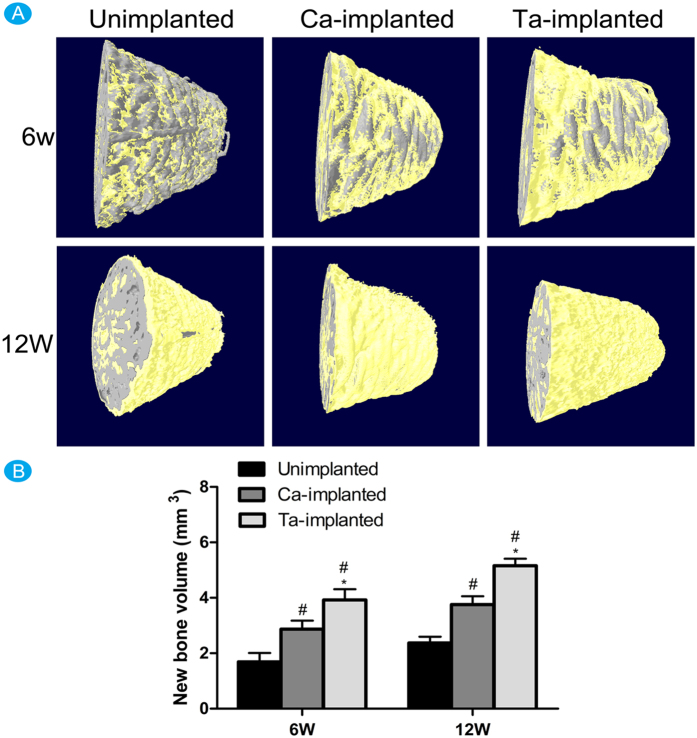
(**A**) The 3D images showing the new bone formation around the surface of different EPT implants at 8 and 16 weeks, respectively. (**B**) Histogram showing the new bone volume around the surface of different EPT implants at 8 and 16 weeks, respectively. ^#^(p < 0.05) when compared with Unimplanted group; *(p < 0.05) when compared with Ca-implanted group.

After 6 and 12 weeks of implantation, micro-CT images of transverse sections along the long axis of EPT implants were employed to evaluate the surface osseointegration of different ETP implants, and the 3D images reconstructed by micro-CT were shown in Fig. 4A. The gray color in images represented EPTs, and the yellow represented the new bone around implants. Obviously, Ta-implanted EPT had the best effect, and Ca-implanted EPT also had significant role in promoting new bone formation around implant at 6 and 12 weeks. According to the statistical analysis, the new bone volume of both Ca-implanted (6w: 2.87 ± 0.31; 12w: 3.76 ± 0.30) and Ta-implanted (6w: 3.92 ± 0.39; 12w: 5.16 ± 0.25) groups was significantly bigger than that of unimplanted (6w: 1.69 ± 0.32; 12w: 2.37 ± 0.23) group (Fig. 4B). Obviously, Ta-implanted EPT group had the biggest new bone volume in our study. To sum up, both Ca- and Ta-implanted EPTs have positive roles in promoting surface osseointegration, but Ta-implanted EPT has the highest effect.

#### Sequential fluorescent labeling analysis

**Figure 5:**
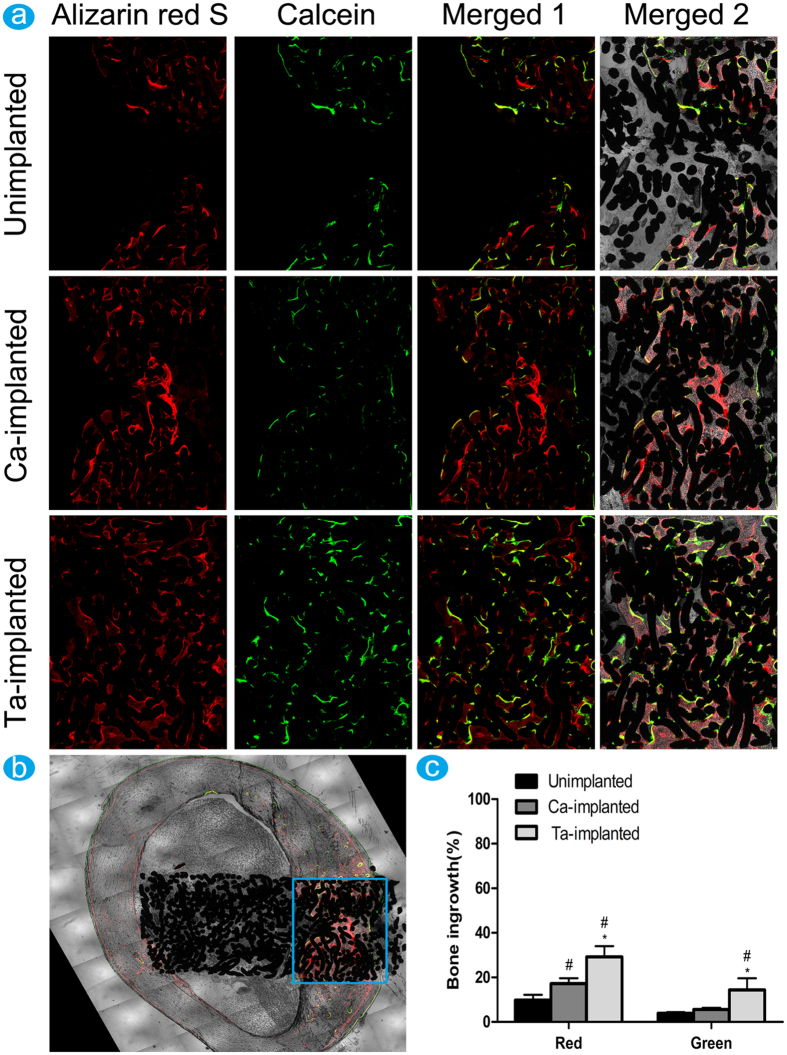
Sequential fluorescence labeling observation: (**a**) red fluorescence (labeling by Alizarin red S) represented the new bone formation at 6 weeks, and green fluorescence (labeling by calcein) represented the new bone formation at 12 weeks, and images were merged in the third row (merged 1). Images in the last row (merged 2) were the fused images of in the third row and EPT implants. (**b**) Illustration of selected area to evaluate new bone formation process. (**c**) Histogram showing the percentage of different color area stained by fluorochromes. ^#^(p < 0.05) when compared with Unimplanted group; *(p < 0.05) when compared with Ca-implanted group.

Polychrome sequential fluorescent labeling was used to evaluate the osteogenic activity. The fluorochrome stained bone at the implant-bone contact segment, which marked with the blue rectangle region, was used to analyze new bone formation activity (Fig. 5b). The fluorescence intensity of both Ca- and Ta-implanted groups was much stronger than that of unimplanted group at 6 weeks. Ta-implanted group had the highest fluorescence intensity at 12 weeks, while no obvious difference was observed between unimplanted and Ca-implanted groups (Fig. 5a). The percentage of different color area clearly revealed the influences of different implants on osteogenic activity (Fig. 5c). At 6 weeks, the percentage of Alizarin red S (red) of both Ta-implanted (29.17 ± 4.83%) and Ca-implanted (17.18 ± 2.45%) groups was significantly higher than that of unimplanted (9.82 ± 2.37%) group. At 12 weeks, Ta-implanted group (14.39 ± 5.24%) had the highest percentage of calcein labeling area (green), while no statistically significant difference existed in unimplanted (3.84 ± 0.55%) and Ca-implanted (5.60 ± 0.81%) groups. The percentage of fluorochrome stained bone approved that Ta-implanted EPT can efficiently promote osteogenic activity at all time points (6 weeks, 12 weeks), while Ca-implanted EPT only has favorable effect at the early stage (6 weeks). These results suggested the positive influence of Ca- and Ta-implanted EPTs in promoting osteogenic activity, and the much more stable and continuous effect of Ta-implanted EPT in the long term.

#### Histomorphometric examination

**Figure 6:**
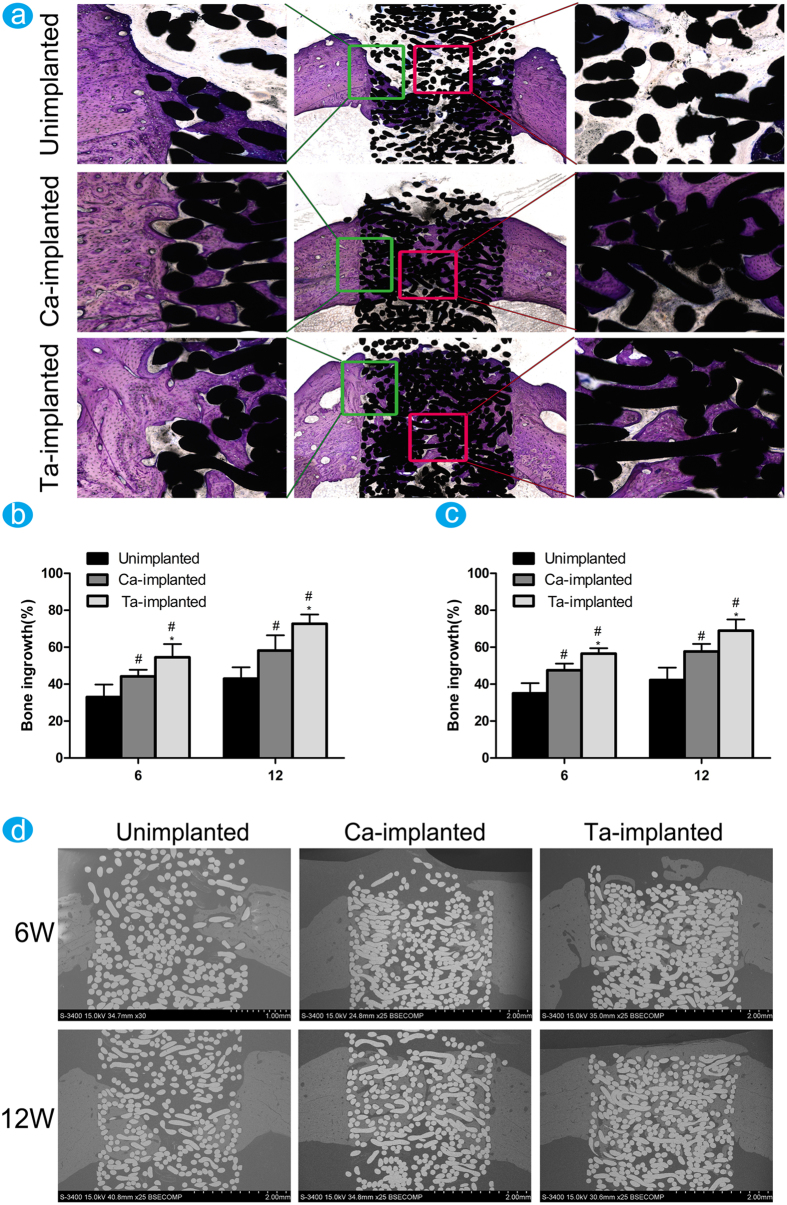
The histological observations and histomorphometric measurements evaluated the variance of new bone ingrowth. (**a**) Undecalcified sections of samples were stained with toluidine blue at 12 weeks. Partial magnifications of the green and red rectangle areas were displayed on the left and right panel, respectively. The percentage of new bone ingrowth and pores in different EPT implants measured from toluidine blue staining (**b**) and back scattered SEM images (**c**) at 6 and 12 weeks. ^#^(p < 0.05) when compared with Unimplanted group; *(p < 0.05) when compared with Ca-implanted group. (**d**) The back scattered SEM images of new bone around and inside pores of EPT implants at 6 and 12 weeks.

The new bone around and inside pores of EPT implants was stained using toluidine blue after implantation for 6 and 12 weeks, and Fig. 6a showed the toluidine blue stained images at 12 weeks post-operation. The partial magnifications of the green rectangle area showed that only a small amount of new bone grew into the pores of unimplanted EPT. Therefore, distinct gap between new bone and implant was observed in experiments. New bone formation in pores could be observed in Ca-implanted EPT, while the gap between bone and metal fibers was distinct in some areas. In comparison, lots of new bone appeared in pores of Ta-implanted EPT. The bone trabecula in Ta-implanted group grew much thicker than that of the other two groups and new bone tightly grew surrounding the Ti fibers without infiltration of fibrous tissue. Meanwhile, the partial magnifications of the red rectangle area showed that no new bone existed in the central core of unimplanted EPT implant, and some new bone appeared in the central core of Ca-implanted EPT implant. In comparison, the central core of Ta-implanted EPT had complete bone ingrowth, and some of new bone began to turn into mature bone matrix. Statistical analysis about new bone ingrowth was performed by comparing the new bone area with the porosity in the selected metal implant area (Fig. 6b). The percentage of both Ca-implanted (6w: 47.51 ± 3.63%, 12w: 57.72 ± 4.11%) and Ta-implanted (6w: 56.52% ± 2.90%, 12w: 68.98 ± 6.04%) groups was significantly higher than that of unimplanted (6w: 35.08 ± 5.48%, 12w: 42.18 ± 6.81%) group, but Ta-implanted EPT had the best effect. Therefore, both Ca- and Ta-implanted EPTs can promote new bone ingrowth, but Ta-implanted EPT has the best effect.

The black scattered SEM images clearly showed the new bone inside the pores of EPT implants at 6 and 12 weeks post-operation (Fig. 6d). It was clear that many intermetallic pores of unimplanted EPT had no bone ingrowth at both time points. Although new bone grew into the pores of Ca-implanted EPT, the bone grew from different sides failed to join together and the gaps were obvious at 12 weeks. In comparison, lots of new bone appeared and converged together in Ta-implanted group at 6 weeks, and only a small area of implant had no bone filling. And sufficient new bone appeared in Ta-implanted EPT at 12 weeks, and the thickness of new bone was consistent with the cortical bone around implant. The new bone ingrowth of both Ca-implanted (6w: 44.17 ± 3.58, 12w: 58.21 ± 8.24) and Ta-implanted (6w: 54.54 ± 7.13, 12w: 72.68 ± 5.02) groups, observed by black scattered SEM, was significantly higher than that of unimplanted (6w: 33.05 ± 6.73, 12w: 42.92 ± 6.23) group (Fig. 6c), but Ta-implanted EPT had the best effect at both time points, which was highly consistent with the new bone stained using toluidine blue. In conclusion, both Ca- and Ta-implanted EPTs can efficiently promote new bone ingrowth, but Ta-implanted EPT has the best influence. And Ta-implanted EPT exhibited much more stable and continuous effects in the long run.

#### Biomechanical push-out test

**Figure 7:**
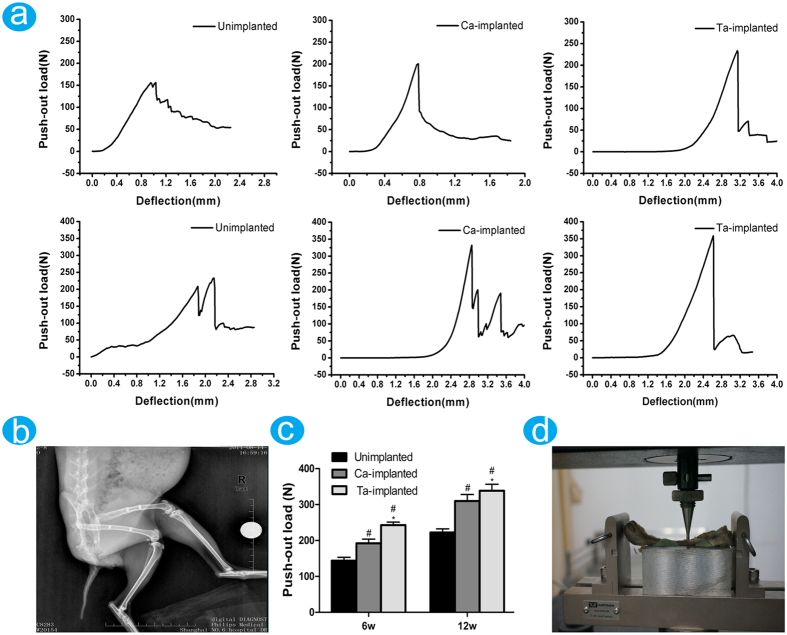
Biomechanical tests: (**a**) the push-out curves of samples at 6 and 12 weeks. (**b**) X-ray image of EPT samples implantation model. (**c**) Histogram showing the failure load peaks of different samples at 6 and 12 weeks. ^#^(p < 0.05) when compared with Unimplanted group; *(p < 0.05) when compared with Ca-implanted group. (**d**) The process of push-out tests.

The X-ray image showed the implantation model of EPT implants (Fig. 7b). The fixation strength between femur and implant was evaluated by push-out tests at 6 and 12 weeks post-operation (Fig. 7a). The process of biomechanical push-out test was shown in Fig. 7d. The fixation strengths of both Ca-implanted (6w: 192.24 ± 11.56N, 12w: 309.86 ± 18.30N) and Ta-implanted (6w: 242.96 ± 8.09N, 12w: 338.64 ± 17.98N) groups were higher than that of unimplanted (6w: 143.72 ± 9.47N, 12w: 222.02 ± 10.58N) group (Fig. 7c), but the Ta-implanted EPT had the highest fixation strength at both time points. These effects further approved the positive roles of Ca- and Ta-implanted EPTs in surface osseointegration and new bone ingrowth, and the highest effects of the developed Ta-implanted EPT.

## Discussions

Although the biologically inactive Ti has good biocompability, its osteoinductive activity is limited^44^,^45^,^46^. Therefore, surface modifications are used to improve the biological properties of porous Ti implants^47^,^48^,^49^,^50^. In the current study, Ca and Ta ions were implanted into the surface layer of EPT by PIII&D method, respectively. And no difference about surface topography and mechanical properties of Ca- and Ta-implanted EPTs was found compared with that of unimplanted EPT. Therefore, these modified EPTs can be reconstructed or reshaped according to the mechanical and clinical demands of bone structures without worrying about the unfavorable changes of base materials in mechanical properties.

*In vitro* and *in vivo* experiments were conducted to investigate the properties of modified EPTs in promoting surface osseointegration and bone ingrowth. Significantly upregulated level of CCK-8, ALP activity, and the concentration of Ca^2+^ in Ca- and Ta-implanted groups (Fig. 2a–c) indicated the positive effects of surface modification on MG-63 cell viability, proliferation, differentiation, and mineralization of ECM. And then osteogenic related markers were further investigated on genetic and protein levels. ALP and Col1a1 are regarded as the early and mature markers of ECM^51^,^52^,^53^,^54^, OSX, BSP are highly relevant with the mineralization of ECM, and OPN and OC play important roles in the early and late stage of osteoblast differentiation^55^,^56^,^57^. Although the detailed mechanisms are not yet understood, the upregulated expression of these genes and proteins (Figs 2d,e and 3) indicate that both Ca- and Ta-implanted EPT can promote osteoblasts differentiation and ECM mineralization at the early age. In addition, although no significant difference was observed in expression of gene Col1a1, the expression of gene ALP, OPN, BSP, OC of Ta-implanted group was significantly higher than that of Ca-implanted group on day 8 and 16 (Fig. 2d,e). The expression of protein OC and OSX of Ta-implanted group was also higher than that of Ca-implanted group on day 8 and 16 (Fig. 3). Therefore, the highly consistent results on molecular, genetic, and protein levels suggested the promoting roles of both Ca- and Ta-implanted EPTs in MG-63 cell viability, proliferation, differentiation and mineralization of ECM. More importantly, Ta-implanted EPT may have more stable and continuous effects in the long term.

In our rabbit femur models, the results of micro-CT tested the beneficial roles of Ca-implanted EPT and the superior effect of Ta-implanted EPT in promoting new bone formation around implant. The results of histomorphometric examinations including toluidine blue staining and back scattered SEM approved the favorable roles of Ca-implanted EPT and excellent effect of Ta-implanted EPT in promoting new bone ingrowth. Therefore, both Ca- and Ta-implanted EPTs were demonstrated to have good effects in promoting bone reconstruction. The osteogenic activity, which was evaluated by sequential fluorescent labeling, was only significantly upregulated at 6 weeks in Ca-implanted group. In contrast, the excellent and highly consistent effects of Ta-implanted EPT at both 6 and 12 weeks suggested the stable and continuous effects in promoting surface osseointegration and new bone ingrowth. The fixation strength of both Ca- and Ta-implanted EPTs, which was investigated by push-out tests, was significantly enhanced compared with that of unimplanted EPT. Since no difference in spatial and mechanical properties was found among three types of EPT implants, the improved bonding strength in enhancing osseointegration and new bone ingrowth may be contributed to the surface modified porous EPT. In addition, more direct contact between new bone and Ta-implanted EPT fibers, which was observed by histomorphometric observation, can partially explain the highest fixation strength of Ta modified EPT implant in our study.

In our previous study, the stable Ta_2_O_5_ on the modified surfaces of PEEK by PIII&D method was observed, and no Ta release was detected in experiments^23^. In present study, the protective film Ta_2_O_5_ was also observed, and it provided large surface area and high activity to generate Ta-OH groups, thus enhancing the bioactivity of Ta-implanted EPT. Ca has been widely studied for the active effects in promoting cell adhesions, proliferation, differentiation, and osseointegration^27^,^58^. However, the corrosion resistance of Ca ion coatings was suspected by researcher recently^59^. The surface charge of Ti implants, local pH and ion concentration may change with the continuing loss of Ca ions, thus resulting in adverse influence on cellular activity^60^. In the present study, both Ca- and Ta-implanted EPTs had favorable properties in promoting osteogenesis. However, Ca-implanted EPT concentrated these favorable effects only at the early age of bone reconstruction and could not reach the higher level of Ta-implanted EPT. In comparison, the Ta-implanted EPT possessed more stable and continuous effects in the long term. Since no difference about surface topography exists in Ca- and Ta-implanted EPT materials, it is reasonable to speculate that differences about osteogenesis between Ca- and Ta-implanted EPTs may be attributed to the loss of Ca ions.

## Conclusions

In this study, Ta-implanted EPT, which combined both the advantages of Ti and Ta, was produced by PIII&D method. *In vitro* experiments approved the positive effects of Ca-implanted EPT and the superior effects of Ta-implanted EPT in improving MG63 cell viability, proliferation, differentiation, mineralization of ECM, and the expression of osteogenic-related markers. *In vivo* experiments demonstrated the favorable properties of Ca-implanted EPT and the excellent properties of Ta-implanted EPT in improving osteogenic activity, surface osseointegration, and new bone ingrowth. The strong bonding strength between host bone and implants, that tested by push-out tests, further approved the helpful effects of Ca-implanted EPT and the high properties of Ta-implanted EPT in improving the quality of osteogenesis. Although Ca-implanted EPTs can efficiently promote surface osseointegration and new bone ingrowth, Ta-implanted EPT has more stable and continuous effects in the long term. Therefore, Ta-implanted EPT can be developed as one of the highly efficient graft materials and has great potential use in bone reconstruction.

## Materials and Methods

### Material synthesis and preparation

The porous titanium was constructed with Ti metal wires (diameter: 0.1 mm) through winding spiral coil, winding, and stamping (fabricated and provided by State Key Lab of Metal Matrix Composites, School of Materials Science and Engineering, Shanghai Jiao Tong University). Cylindrical samples were prepared in different dimensions for mechanical property evaluation, *in vitro* and *in vivo* experiments (Table 3). Prior to PIII&D treatment, EPTs were sequentially washed in an ultrasonic cleaner by acetone, ethanol and ultra-pure water for 10 minutes. Ca or Ta ions were implanted into EPTs using a filtered cathodic arc source that housed a 99.99% pure Ca or Ta rod with a diameter of 10 mm, respectively. The chamber was evacuated to a pressure of 5 × 10^−3^ Pa before PIII&D. Then EPTs were washed with argon (Ar) for 15 minutes at a flow rate of 5 sccm (standard cubic centimeter per minute) in order to attain stable ionization of Ca or Ta, and fixed on a rotating sample stage and connected to a high voltage. Ca or Ta ions were implanted by applying a pulsed negative high voltage, and the sample stage was continuously rotated to obtain uniform ion implantation. Table 4 listed the significant parameters with corresponding sample designations of PIII&D.

### Surface structure and chemical characterization

Field emission scanning electron microscopes (Hitachi SN-3400, Hitachi, Tokyo, Japan) were used to determine surface morphology, and the acceleration voltage was set at 15 kV. The surface chemical states were examined by XPS (ESCALAB250, Thermo Fisher Scientific Inc., MA, USA).

### Mechanical property evaluation

Compressive yielding stress and Young’s modulus were determined using an electronic universal material testing machine (Zwick T1-FR020.A50, Instron, MA, USA). The precision of sensor was 2 kN, and the moving speed of cross-head was 0.6 mm/min.

### *In vitro* studies

#### MG-63 cell culture

MG-63, a cell line from human osteosarcoma, was obtained from the cell bank of Institute of Biochemistry and Cell Biology, SIBS, CAS (Shanghai, China). Cells were cultured using DMEM (Thermo Fisher Scitific Inc.) supplemented with 10% (v/v) fetal calf serum (FBS) (Thermo Fisher Scitific Inc.), 100 U/mL Penicillin (Thermo Fisher Scitific Inc.), 100 μg/ml Streptomycin (Thermo Fisher Scitific Inc.) in a humid and 5% CO_2_ incubator at 37 °C. Cells at log phase were prepared into suspensions and used for *in vitro* experiments.

#### CCK-8 assay

The cell counting kit-8 (CCK-8) (Dojindo, Kumamoto-ken, Japan) assays were conducted to quantitatively determine MG-63 cell viability and proliferation. 1 × 10^4^ cells in 1,500 μl culture medium per well was added to a 24-well-plate that placed with different EPT samples. After cultivation for 8 hours, EPT samples were moved to a new culture plate and further incubated in 500 μl culture medium per well for 1, 4, 8, and 16 days. 50 μl CCK-8 solutions was added to culture medium (500 μl) and incubated for 4 hours. 100 μl supernatants per well were transferred to a 96-well-plate. The cell viability was expressed as the optical density (OD) values at 450 nm and determined by an enzyme-linked detector (Thermo Fisher Scientific Inc.).

#### Measurement of intracellular Ca ions

1 × 10^4^ cells per well was seeded in a 24-well-plate that placed with EPT samples. After cultivation for 8 hours, EPT samples were moved to a new culture plate and further incubated in culture medium for 8 and 16 days. Then MG-63 cells were collected and washed in Dulbecco’s Phosphate Buffered Saline (dPBS) for three times. After incubation with 5 μM Fluo 3-AM (DOJINDO, Kumamoto-ken, Japan) for 30 minutes, cells were gently rinsed in D-Hanks’ solution for three times and re-suspended in Krebs-Ringer-HEPES (KRH) buffer. The concentration of Ca ions was determined based on the absorbance at 490 nm through flow cytometry (FACS Calibur, BD, NJ, USA).

#### Alkaline phosphate (ALP) activity test

MG-63 cell differentiation was evaluated by measuring ALP activity. MG-63 cells were seeded at a density of 1 × 10^4^ cells per milliliter in a 24-well-plate that placed with different EPT samples. After cultivation for 8 hours, EPT samples were moved to a new culture plate and further incubated for 8 and 16 days. The cells were lysed in 0.1% Triton X-100 and then incubated with *p*-nitrophenyl phosphate (*p*NPP) (Sigma-Aldrich) at 37 °C. After incubation for 60 minutes, ALP activity was determined based on the OD values at 405 nm. The total protein content was determined using the BCA protein kit and the ALP activity was normalized to the total protein synthesis and expressed as nmol/min/mg total proteins.

#### Quantitative real time polymerase chain reaction (qRT-PCR) analysis

MG-63 cells were seeded at a density of 1 × 10^4^ cells per milliliter in cell culture plate that placed with different EPT samples. After cultivation for 8 hours, EPT samples were moved to a new culture plate and further incubated for 8 and 16 days. TRIzol reagent (Thermo Fisher Scientific Inc.) was used to extract total RNA according to the manufacturer’s instructions. The quality of RNA was assessed depending on 1.5% agarose gel electrophoresis, and the RNA concentration was determined by Heλiosγ (Thermo Fisher Scientific). 1 μg of total RNA was used to synthesize cDNA using the PrimeScript™ RT reagent Kit (Takara). The expression level of osteogenic-related markers, including collagen, type I, alpha 1 (Col1a1), ALP, osteopontin (OPN), bone sialoprotein (BSP), osteocalcin (OC), osterix (OSX) were determined by qRT-PCR using SYBR^®^ Premix Ex Taq^TM^ kit (Takara) according to the manufacturer’s instructions. Results were normalized to β-actin and expressed as relative expression level of unimplanted group using *2*^−ΔΔ*Ct*^ method. PCR primer sequences were listed in Table 5.

#### Western blotting

After cultivation with different EPTs for 8 and 16 days, the total protein was extracted from the cells using M-PER protein extraction reagent (Pierce). The protein concentration was measured by BCA Protein Assay Kit (Pierce) according to the manufacturer’s instructions. Proteins were run on SDS-PAGE gel and transferred in to a PVDF membrane (Millipore, MA, USA) according to the standard protocol. After blocking in 5% skim milk for 2 hours at room temperature, the membrane was incubated with primary antibodies against Col1a1, OPN, BSP, OC and OSX (Santa Cruz Biotech) overnight at 4 °C, and then incubated in the solution of secondary antibodies for 1 hour at room temperature. β-actin (Santa Cruz Biotech) was used as the internal control. Protein bands were captured using ECL detection kit (Pierce).

### *In vivo* evaluation of surface osseointegration and bone ingrowth

#### Surgical procedures

Four male New Zealand rabbits per group, that aged 20 weeks old, were used in study. The experimental protocol was approved by the Animal Care and Experiment Committee of the Sixth People’s Hospital Affiliated to Shanghai Jiao Tong University (Shanghai, China). After adaptive breeding for 2 weeks, rabbits were administrated using 20 mg/kg cefazolin by preventive injection half hours before surgery. Then animals were hocused by injection of 25 mg/kg Phenobarbital via marginal ear vein. Rabbits were fixed on surgical table, and both legs were shaved and sterilized. Then the mid-shaft of both left and right femur was exposed by a lateral longitudinal skin incision. Two cylindrical holes (2.95 mm in diameter) with a distance of 20 mm were drilled through the lateral cortical bone of femur, and the cylindrical EPT implants (3mm in diameter) were bilaterally press-fitted into the holes (Fig. 7b). Then the surgical wound was sutured carefully.

#### Sequential fluorescent labeling

A polychrome sequential fluorescent labeling method was used to study osteogenic activity. Fluorochromes were administered intraperitoneally at a sequence of 30 mg/kg alizarin red S (Sigma-Aldrich) at 6 weeks, and 20 mg/kg calcein (Sigma-Aldrich) at 12 weeks. Images were captured using a laser scanning confocal microscope (Carl Zeiss LSM 5 PASCAL, Zeiss, Jena, Germany).

#### Sample preparation

After implantation for 6 and 12 weeks, two experimental rabbits of each group were executed, and the bilateral femurs were completely took out. EPT implants in two experimental rabbits of each group were divided into eight samples: four random samples were prepared for biomechanical tests, and the other four samples were fixed with 10% formalin for micro-CT tests and histomorphometric examinations.

#### Micro-CT assay

New bone formation around the surface of implants was imaged by micro-CT (Micro CT Skyscan 1176, Bruker BioSpin, Belgium). The scanning parameters were set at 45 kV, 550 μA, and 3000 ms with a resolution of 9 μm. After scanning, the three-dimensional (3D) models were reconstructed using the NRecon (Skyscan Company, Bruker micro CT, Belgium), and the 3D models were generated with CTVol (Skyscan Company, Bruker micro CT). New bone volume was measured using DataViewer software (SkyScan Company) and CTAn program (SkyScan Company).

#### Push-out test

The biomechanical tests were conducted using a universal material testing machine (Instron 5569, Instron, MA, USA). A custom made holder was used to fix testing samples to ensure the test load consistent with the long axis of EPT implant, and the fissure between sample and holder was filled by bone cement (Fig. 7d). A special designed crosshead with the same diameter as EPT implants was prepared to apply load on implants. Push-out tests were performed at a loading rate of 5 mm/min. The load-displacement curve was recorded by time, and the failure load was determined as the peak load value of the push-out load curve.

#### Histomorphometric examination

Immediately after micro-CT assays, four femur samples of each group were dehydrated by ascending concentrations of ethanol and embedded in polymethylmetacrylate (PMMA). The embedded specimens were cut into 150 μm thick sections along the long axis of implants using a Leica SP1600 saw microtome (Leica, Solms, Germany). The sections were then ground and polished to a final thickness of 40 μm (Exakt, Norderstedt, Germany) for fluorescence labeling observation. Excitation/emission wavelengths of chelating fluorochromes were 543/617 nm for Alizarin Red S (red) and 488/517 nm for calcein (green). After fluorescence labeling observation, slices were stained with toluidine blue and examined the new bone tissue around the surface and inside pores of implants. The images were captured by an optical microscope (Olympus, Tokyo, Japan). Finally, back scattered scanning electron microscopy (SEM) was used to determine the percentage of new bone ingrowth inside the implants of the sections. The area for bone ingrowth measurement within each implant was shown as Fig. 5b.

#### Statistical analysis

Images from sequential fluorescent labeling, optical microscopy and back scattered SEM were analyzed using the Image-Pro Plus 6.0 (Media Cybernetic, Silver Springs, MD, U.S.A.). All data were presented as *mean* ± *standard deviation (SD)*. One-way ANOVA and SNK post hoc based on the normal distribution and equal variance assumption test were adopted in statistical comparisons. All statistical analysis was determined using SPSS16.0 (IBM, New York, U.S.A.). The differences were considered statistically significant at ^#^(*p* < *0.05*) when compared with unimplanted EPT; ^*^(*p* < *0.05*) when compared with Ca-implanted EPT.

### Ethics statement

All animal experiments in this study were approved by the Animal Care and Experiment Committee of the Sixth People’s Hospital Affiliated to Shanghai Jiao Tong University. All procedures were carried out in accordance with the approved guidelines.

## Additional Information

**How to cite this article**: Wang, Q. *et al*. Tantalum implanted entangled porous titanium promotes surface osseointegration and bone ingrowth. *Sci. Rep.* **6**, 26248; doi: 10.1038/srep26248 (2016).

## Figures and Tables

**Table 1:** Elemental analysis of EPT surface.

**Elements**	**Unimplanted EPT**	**Ca-implanted EPT**	**Ta-implanted EPT**
Ti	23.07%	16.11%	17.07%
O	76.93%	66.30%	71.97%
Ca	–	17.59%	–
Ta	–	–	10.96%

**Table 2:** The mechanical performances of EPTs.

**EPTs**	**Yielding stress (MPa)**	**Young’s modulus (MPa)**
Unimplanted EPT	7.27 ± 0.31	114.30 ± 5.86
Ca-implanted EPT	7.20 ± 0.26	119.00 ± 2.65
Ta-implanted EPT	7.37 ± 0.57	117.30 ± 11.37

**Table 3:** Applications and parameters of cylindrical EPT samples.

**EPT samples**	**Diameter**	**Height**	**Applications**
EPT1	10 mm	20 mm	Mechanical property tests
EPT2	3 mm	10 mm	*In vivo* animal experiments SEM observation
EPT3	10 mm	2 mm	X-ray photoelectron spectroscopy (XPS) analysis *In vitro* experiments

**Table 4:** Main parameters of PIII&D.

	**Unimplanted EPT**	**Ca-implanted EPT**	**Ta-implanted EPT**
Voltage pulse duration (μs)	–	500	500
Pulsing frequency (Hz)	–	5	5
Ion implantation voltage (kV)	–	15	15
Ion implantation time (h)	–	2	2
Pressure (Pa)	–	2.5 × 10^−3^	2.5 × 10^−3^

**Table 5:** Primer pairs used in qRT-PCR analysis.

**Gene**	**Primers (F = forward, R = reverse)**	**Amplicon**
Col1a1	F: ACCTCCGGCTCCTGCTCCTCTTAG	235
	R: GCGCCGGGGCAGTTCTTGGTCT	
ALP	F: GACAATCGGAATGAGCCCACAC	222
	R: GTACTTATCCCGCGCCTTCACCAC	
OPN	F: TCTGATGAATCTGATGAACTGGTC	195
	R: GGTGATGTCCTCGTCTGTAGCA	
BSP	F: AAGAAGGGGAAGAAGAAAGTGTCA	218
	R: GTATTCATTGGCGCCCGTGTATTC	
OC	F: AGCCCAGCGGTGCAGAGTCCA	224
	R: GCCGTAGAAGCGCCGATAGG	
OSX	F: GCTGCCCACCTACCCATCTGACTT	207
	R: CTGCCCCCATATCCACCACTACCC	
β-actin	F: CCCAAGGCCAACCGCGAGAAGATG	219
	R: GTCCCGGCCAGCCAGGTCCAGA	
